# (15-Crown-5-κ^5^ *O*)[*S*-(*E*)-1,2-dichloro­vinyl thio­sulfato-κ*O*]sodium

**DOI:** 10.1107/S1600536811022252

**Published:** 2011-06-18

**Authors:** Dong-Qing Sun, Jing-Kui Yang

**Affiliations:** a College of Chemistry and Chemical Engineering, Graduate University of Chinese Academy of Sciences, Beijing 100049, People’s Republic of China

## Abstract

In the title complex, [Na(C_2_HCl_2_O_3_S_2_)(C_10_H_20_O_5_)], there are two independent complex units in the asymmetric unit, one of which has a 55:45% disorder in the 15-crown-5 component. The coordination sphere about the Na atom in each complex unit comprises five bonds to O atoms of the crown ether [Na—O = 2.390 (7)–2.466 (6) Å] and one to a thio­sulfate O atom [Na—O = 2.305 (4) and 2.447 (3) Å].

## Related literature

For the usage of sodium alkyl thio­sulfate in synthesis, see: Crich *et al.* (2007 ▶); Cruz *et al.* (2001 ▶); Guth *et al.* (1979 ▶); Huang *et al.* (1997 ▶); Wille *et al.* (1977 ▶). For the crystal structure of similiar 15-crown-5 complexes, see: Blais *et al.* (2001 ▶); McIntosh *et al.* (2001 ▶).
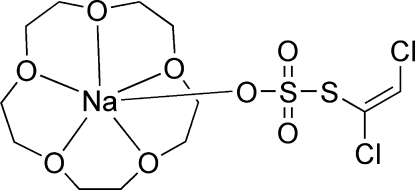


## Experimental

### 

#### Crystal data

[Na(C_2_HCl_2_O_3_S_2_)(C_10_H_20_O_5_)]
*M* *_r_* = 451.30
Triclinic, 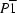

*a* = 8.4455 (18) Å
*b* = 15.787 (4) Å
*c* = 16.778 (4) Å
α = 110.854 (19)°
β = 99.53 (2)°
γ = 100.76 (2)°
*V* = 1987.0 (9) Å^3^
*Z* = 4
Mo *K*α radiation
μ = 0.59 mm^−1^
*T* = 295 K
0.50 × 0.40 × 0.30 mm

#### Data collection

Bruker *P*4 four-circle diffractometer
Absorption correction: empirical (using intensity measurements) (North *et al.*, 1968 ▶) *T*_min_ = 0.465, *T*_max_ = 0.506
8439 measured reflections
6904 independent reflections
5027 reflections with *I* > 2σ(*I*)
*R*_int_ = 0.027
3 standard reflections every 97 reflections  intensity decay: none

#### Refinement

*R*[*F* ^2^ > 2σ(*F* ^2^)] = 0.059
*wR*(*F* ^2^) = 0.120
*S* = 1.07
6904 reflections
587 parameters
52 restraints
H-atom parameters constrained
Δρ_max_ = 0.62 e Å^−3^
Δρ_min_ = −0.46 e Å^−3^

### 

Data collection: *XSCANS* (Bruker, 1997 ▶); cell refinement: *XSCANS*; data reduction: *XSCANS*; program(s) used to solve structure: *SHELXS97* (Sheldrick, 2008 ▶); program(s) used to refine structure: *SHELXL97* (Sheldrick, 2008 ▶); molecular graphics: *SHELXTL* (Sheldrick, 2008 ▶); software used to prepare material for publication: *SHELXTL*.

## Supplementary Material

Crystal structure: contains datablock(s) I, global. DOI: 10.1107/S1600536811022252/zs2116sup1.cif

Structure factors: contains datablock(s) I. DOI: 10.1107/S1600536811022252/zs2116Isup2.hkl

Additional supplementary materials:  crystallographic information; 3D view; checkCIF report

**Fig. 1.**
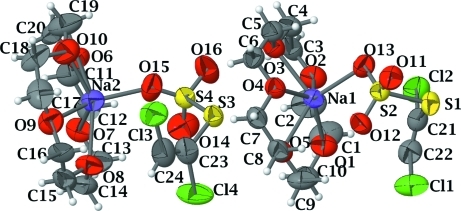
Molecular structure and atom labeling scheme for the two independent molecules of the title compound in the asymmetric unit. Atoms of the minor component of the 55/45% disordered 15-crown-5 ligand associated with Na2 have been omitted. The displacement ellipsoids are drawn at the 35% probability level.

Enhanced figure: interactive version of Fig. 1

## Figures and Tables

**Fig. 2.**
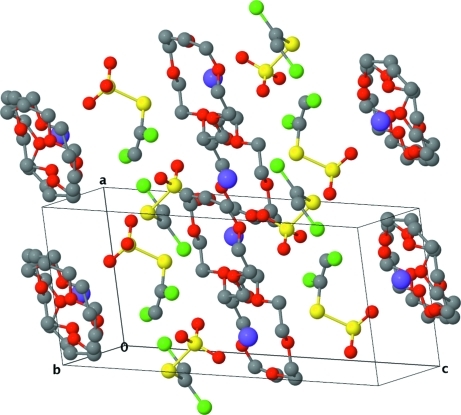
Molecular packing of the title compound.

## supplementary crystallographic information

### Comment

Sodium alkyl thiosulfates are a class of useful synthetic intermediates, and are widely used in the construction of C—S, S—S, C—N, C—O and S—N bonds (Crich *et al.*, 2007; Cruz *et al.*, 2001; Guth *et al.*, 1979; Huang *et al.*, 1997; Wille *et al.*, 1977). However, most of these reactions are carried out under vigorous chemical conditions. When the alkyl chain is short, the reaction can be only carried out in highly polar solvents such as water and methanol, due to the restricted solubility of the products. These factors restrict the applications of sodium alkyl thiosulfates, especially thermolabile compounds. In addition, most groups associated with the sulfur atoms are saturated whereas research on sodium olefinic thiosulfate has rarely been reported. We synthesized the title compound C_12_H_21_Cl_2_NaO_8_S_2_ (I) by reaction of sodium thiosulfate with trichloroethylene and 15-crown-5 in acetonitrile at room temperature. Compound (I) showed significantly improved solubility in some moderately polar solvents such as ethyl acetate, dichloromethane, acetone and toluene.

In the structure of (I) there are two independent complex units in the asymmetric unit (Fig. 1), one of which (involving Na2) has a 55/45% disorder in the 15-crown-5 component. The coordination sphere about the Na centre in each complex unit comprises five bonds to O atoms of the crown ether [Na—O range 2.390 (7)–2.466 (6) Å] and one to a thiosulfato O donor [Na1—O13, 2.447 (3) and Na2—O15, 2.305 (4) Å]. There is a longer Na1···O12 (thiosulfate) contact in one of the complex units. The vinyl chain conformation of the two (*E*)-dichlorovinylsulfato ligands is similar [torsion angles S2—S1—C21—C22, -88.8 (5)°; S4—S3—C23—C24, -96.8 (6)°].

### Experimental

Ground Na_2_S_2_O_3_ . 5H_2_O (0.496 g, 2 mmol) and 15-crown-5 (0.441 g, 2 mmol) were suspended in 40 ml of acetonitrile. Trichloroethylene (0.526 g, 4 mmol) and NaOH (0.08 g, 2 mmol) were added with stirring which was continued for 72 h at room temperature. The reaction product was filtered and washed with acetonitrile and after removal of solvent, the products were separated using silica gel column chromatography to give the title compound in 51% yield. Crystals were obtained by diffusing *n*-pentane into a concentrated solution of the compound in acetone at room temperature.

### Refinement

One of the 15-crown-5 components, coordinated to Na2, was disordered and occupancies were assigned to be 0.55 and 0.45 in the final refinement. All non-hydrogen atoms were subjected to anisotropic refinement. All hydrogen atoms were generated geometically and were included in the refinement with C—H bond distances of 0.93–0.97 Å and *U*_iso_(H) = 1.2*U*_eq_(C), using a riding model.

### Crystal data

**Table:**

[Na(C_2_HCl_2_O_3_S_2_)(C_10_H_20_O_5_)]	*Z* = 4
*M**_r_* = 451.30	*F*(000) = 936
Triclinic, *P*1	*D*_x_ = 1.509 Mg m^−^^3^
Hall symbol: -P 1	Mo *K*α radiation, λ = 0.71073 Å
*a* = 8.4455 (18) Å	Cell parameters from 38 reflections
*b* = 15.787 (4) Å	θ = 7.2–12.5°
*c* = 16.778 (4) Å	µ = 0.59 mm^−^^1^
α = 110.854 (19)°	*T* = 295 K
β = 99.53 (2)°	Prism, colorless
γ = 100.76 (2)°	0.50 × 0.40 × 0.30 mm
*V* = 1987.0 (9) Å^3^	

### Data collection

**Table:**

Bruker P4 four-circle diffractometer	5027 reflections with *I* > 2σ(*I*)
Radiation source: fine-focus sealed tube	*R*_int_ = 0.027
graphite	θ_max_ = 25.1°, θ_min_ = 2.3°
ω scans	*h* = −10→1
Absorption correction: empirical (using intensity measurements) (North *et al.*, 1968)	*k* = −17→17
*T*_min_ = 0.465, *T*_max_ = 0.506	*l* = −19→19
8439 measured reflections	3 standard reflections every 97 reflections
6904 independent reflections	intensity decay: none

### Refinement

**Table:**

Refinement on *F*^2^	Primary atom site location: structure-invariant direct methods
Least-squares matrix: full	Secondary atom site location: difference Fourier map
*R*[*F*^2^ > 2σ(*F*^2^)] = 0.059	Hydrogen site location: inferred from neighbouring sites
*wR*(*F*^2^) = 0.120	H-atom parameters constrained
*S* = 1.07	*w* = 1/[σ^2^(*F*_o_^2^) + (0.001*P*)^2^ + 2.5*P*] where *P* = (*F*_o_^2^ + 2*F*_c_^2^)/3
6904 reflections	(Δ/σ)_max_ < 0.001
587 parameters	Δρ_max_ = 0.62 e Å^−^^3^
52 restraints	Δρ_min_ = −0.46 e Å^−^^3^

### Special details

**Table:**

Geometry. All e.s.d.'s (except the e.s.d. in the dihedral angle between two l.s. planes) are estimated using the full covariance matrix. The cell e.s.d.'s are taken into account individually in the estimation of e.s.d.'s in distances, angles and torsion angles; correlations between e.s.d.'s in cell parameters are only used when they are defined by crystal symmetry. An approximate (isotropic) treatment of cell e.s.d.'s is used for estimating e.s.d.'s involving l.s. planes.
Refinement. Refinement of *F*^2^ against ALL reflections. The weighted *R*-factor *wR* and goodness of fit *S* are based on *F*^2^, conventional *R*-factors *R* are based on *F*, with *F* set to zero for negative *F*^2^. The threshold expression of *F*^2^ > σ(*F*^2^) is used only for calculating *R*-factors(gt) *etc*. and is not relevant to the choice of reflections for refinement. *R*-factors based on *F*^2^ are statistically about twice as large as those based on *F*, and *R*- factors based on ALL data will be even larger.

### Fractional atomic coordinates and isotropic or equivalent isotropic displacement parameters (Å^2^)

**Table:**

	*x*	*y*	*z*	*U*_iso_*/*U*_eq_	Occ. (<1)
Na1	0.14966 (18)	0.28710 (10)	0.45817 (10)	0.0609 (4)	
Na2	0.5805 (2)	0.29126 (11)	0.97277 (11)	0.0736 (4)	
S1	−0.10492 (14)	0.25340 (8)	0.18327 (7)	0.0712 (3)	
S2	0.09233 (13)	0.34225 (7)	0.29733 (7)	0.0640 (3)	
S3	0.33363 (13)	0.23429 (8)	0.67908 (7)	0.0670 (3)	
S4	0.23814 (13)	0.21257 (8)	0.78255 (7)	0.0672 (3)	
Cl1	0.0369 (2)	0.06689 (10)	0.14297 (12)	0.1146 (5)	
Cl2	−0.33675 (17)	0.16304 (11)	0.26409 (9)	0.1013 (4)	
Cl3	0.69742 (18)	0.36967 (9)	0.78535 (11)	0.1159 (5)	
Cl4	0.5072 (2)	0.07699 (10)	0.63686 (15)	0.1431 (7)	
O1	0.0689 (4)	0.1173 (2)	0.4015 (2)	0.0860 (9)	
O2	−0.0849 (4)	0.2292 (2)	0.5087 (2)	0.0854 (9)	
O3	0.1483 (3)	0.3996 (2)	0.6056 (2)	0.0766 (8)	
O4	0.4074 (3)	0.41335 (19)	0.53040 (18)	0.0685 (7)	
O5	0.3958 (4)	0.2259 (2)	0.4629 (2)	0.0786 (8)	
O11	0.1432 (4)	0.4189 (2)	0.2724 (2)	0.0852 (9)	
O12	0.2101 (4)	0.2893 (2)	0.3038 (2)	0.0847 (9)	
O13	0.0179 (4)	0.3630 (2)	0.37160 (19)	0.0840 (9)	
O14	0.2177 (6)	0.1163 (3)	0.7663 (3)	0.1230 (14)	
O15	0.3597 (4)	0.2733 (3)	0.8616 (2)	0.0997 (11)	
O16	0.0893 (4)	0.2409 (3)	0.7701 (2)	0.1097 (13)	
C1	−0.0696 (8)	0.0775 (4)	0.4247 (4)	0.123 (2)	
H1A	−0.0516	0.0220	0.4332	0.147*	
H1B	−0.1663	0.0569	0.3756	0.147*	
C2	−0.1070 (8)	0.1365 (4)	0.5015 (5)	0.126 (2)	
H2A	−0.2216	0.1112	0.5003	0.151*	
H2B	−0.0360	0.1363	0.5531	0.151*	
C3	−0.0934 (6)	0.2894 (4)	0.5921 (3)	0.0826 (14)	
H3A	−0.0304	0.2759	0.6379	0.099*	
H3B	−0.2082	0.2803	0.5960	0.099*	
C4	−0.0214 (5)	0.3892 (3)	0.6038 (3)	0.0817 (13)	
H4A	−0.0780	0.4013	0.5554	0.098*	
H4B	−0.0341	0.4331	0.6585	0.098*	
C5	0.2332 (6)	0.4879 (3)	0.6108 (3)	0.0809 (13)	
H5A	0.2289	0.5378	0.6641	0.097*	
H5B	0.1839	0.4991	0.5603	0.097*	
C6	0.4105 (5)	0.4838 (3)	0.6120 (3)	0.0757 (12)	
H6A	0.4787	0.5441	0.6194	0.091*	
H6B	0.4567	0.4683	0.6603	0.091*	
C7	0.5549 (5)	0.3840 (3)	0.5322 (3)	0.0743 (12)	
H7A	0.5894	0.3757	0.5864	0.089*	
H7B	0.6434	0.4310	0.5294	0.089*	
C8	0.5207 (5)	0.2915 (3)	0.4533 (3)	0.0769 (12)	
H8A	0.4837	0.2992	0.3990	0.092*	
H8B	0.6211	0.2701	0.4515	0.092*	
C9	0.3475 (7)	0.1367 (3)	0.3907 (4)	0.0943 (16)	
H9A	0.4395	0.1080	0.3884	0.113*	
H9B	0.3149	0.1438	0.3357	0.113*	
C10	0.2015 (7)	0.0758 (3)	0.4046 (4)	0.0973 (16)	
H10A	0.1694	0.0127	0.3588	0.117*	
H10B	0.2321	0.0718	0.4612	0.117*	
O6	0.4038 (7)	0.3119 (5)	1.0723 (4)	0.0780 (16)	0.55
O7	0.4955 (9)	0.1478 (4)	0.9969 (5)	0.085 (2)	0.55
O8	0.7302 (8)	0.1748 (6)	0.9068 (6)	0.094 (3)	0.55
O9	0.8806 (8)	0.3433 (5)	1.0235 (5)	0.100 (2)	0.55
O10	0.6642 (9)	0.4528 (5)	1.0732 (5)	0.111 (2)	0.55
C11	0.3517 (17)	0.2311 (6)	1.0891 (9)	0.092 (3)	0.55
H11A	0.2451	0.2301	1.1038	0.110*	0.55
H11B	0.4315	0.2349	1.1398	0.110*	0.55
C12	0.3349 (14)	0.1412 (9)	1.0124 (9)	0.083 (4)	0.55
H12A	0.2998	0.0873	1.0265	0.099*	0.55
H12B	0.2542	0.1351	0.9609	0.099*	0.55
C13	0.5037 (15)	0.0754 (7)	0.9207 (8)	0.102 (4)	0.55
H13A	0.4324	0.0765	0.8696	0.123*	0.55
H13B	0.4623	0.0153	0.9237	0.123*	0.55
C14	0.6777 (19)	0.0846 (9)	0.9097 (12)	0.096 (6)	0.55
H14A	0.7502	0.0807	0.9588	0.116*	0.55
H14B	0.6798	0.0351	0.8556	0.116*	0.55
C15	0.9060 (10)	0.1998 (9)	0.9349 (11)	0.090 (4)	0.55
H15A	0.9522	0.1652	0.8882	0.107*	0.55
H15B	0.9413	0.1860	0.9859	0.107*	0.55
C16	0.9630 (12)	0.3044 (8)	0.9581 (8)	0.085 (4)	0.55
H16A	1.0829	0.3278	0.9809	0.102*	0.55
H16B	0.9300	0.3187	0.9071	0.102*	0.55
C17	0.933 (2)	0.4411 (7)	1.0672 (11)	0.108 (5)	0.55
H17A	0.9393	0.4671	1.0230	0.129*	0.55
H17B	1.0449	0.4577	1.1036	0.129*	0.55
C18	0.8304 (10)	0.4900 (9)	1.1248 (8)	0.099 (7)	0.55
H18A	0.8416	0.4775	1.1778	0.118*	0.55
H18B	0.8658	0.5575	1.1418	0.118*	0.55
C19	0.5348 (15)	0.4655 (10)	1.1164 (10)	0.125 (5)	0.55
H19A	0.4417	0.4680	1.0757	0.150*	0.55
H19B	0.5737	0.5265	1.1655	0.150*	0.55
C20	0.470 (2)	0.3941 (8)	1.1513 (8)	0.099 (6)	0.55
H20A	0.5585	0.3869	1.1911	0.118*	0.55
H20B	0.3842	0.4106	1.1812	0.118*	0.55
O6'	0.3803 (12)	0.2310 (8)	1.0416 (6)	0.112 (4)	0.45
O7'	0.5950 (12)	0.1367 (6)	0.9737 (7)	0.108 (3)	0.45
O8'	0.8220 (11)	0.2517 (6)	0.9298 (6)	0.098 (2)	0.45
O9'	0.8282 (9)	0.4264 (6)	1.0408 (6)	0.096 (3)	0.45
O10'	0.5751 (15)	0.4058 (6)	1.1183 (6)	0.130 (4)	0.45
C11'	0.382 (3)	0.1423 (11)	1.0431 (11)	0.106 (8)	0.45
H11C	0.2735	0.1097	1.0441	0.127*	0.45
H11D	0.4639	0.1491	1.0945	0.127*	0.45
C12'	0.4276 (15)	0.0896 (13)	0.9594 (11)	0.107 (6)	0.45
H12C	0.4160	0.0242	0.9501	0.128*	0.45
H12D	0.3580	0.0924	0.9088	0.128*	0.45
C13'	0.643 (2)	0.0950 (14)	0.8934 (13)	0.127 (11)	0.45
H13C	0.5706	0.0979	0.8436	0.153*	0.45
H13D	0.6438	0.0302	0.8808	0.153*	0.45
C14'	0.819 (2)	0.1582 (8)	0.9158 (15)	0.111 (8)	0.45
H14C	0.8849	0.1573	0.9685	0.134*	0.45
H14D	0.8704	0.1330	0.8681	0.134*	0.45
C15'	0.964 (2)	0.3298 (13)	0.9621 (12)	0.182 (14)	0.45
H15C	0.9604	0.3603	0.9211	0.218*	0.45
H15D	1.0624	0.3067	0.9631	0.218*	0.45
C16'	0.9809 (15)	0.4026 (12)	1.0529 (12)	0.099 (6)	0.45
H16C	0.9921	0.3764	1.0971	0.118*	0.45
H16D	1.0751	0.4565	1.0693	0.118*	0.45
C17'	0.812 (3)	0.4761 (18)	1.1276 (9)	0.175 (16)	0.45
H17C	0.9005	0.5344	1.1553	0.210*	0.45
H17D	0.8299	0.4387	1.1617	0.210*	0.45
C18'	0.648 (2)	0.4992 (8)	1.1327 (12)	0.125 (6)	0.45
H18C	0.6494	0.5447	1.1899	0.150*	0.45
H18D	0.6026	0.5171	1.0860	0.150*	0.45
C19'	0.4106 (17)	0.3922 (12)	1.1280 (14)	0.095 (6)	0.45
H19C	0.3372	0.3996	1.0814	0.114*	0.45
H19D	0.4062	0.4367	1.1845	0.114*	0.45
C20'	0.362 (2)	0.2922 (10)	1.1220 (9)	0.105 (5)	0.45
H20C	0.4322	0.2866	1.1708	0.126*	0.45
H20D	0.2470	0.2759	1.1252	0.126*	0.45
C21	−0.1712 (5)	0.1594 (3)	0.2119 (3)	0.0723 (11)	
C22	−0.1165 (6)	0.0862 (3)	0.1972 (3)	0.0857 (14)	
H22A	−0.1623	0.0414	0.2169	0.103*	
C23	0.5175 (6)	0.2014 (4)	0.6936 (3)	0.0930 (15)	
C24	0.6631 (7)	0.2484 (4)	0.7327 (3)	0.1042 (18)	
H24A	0.7502	0.2201	0.7339	0.125*	

### Atomic displacement parameters (Å^2^)

**Table:**

	*U*^11^	*U*^22^	*U*^33^	*U*^12^	*U*^13^	*U*^23^
Na1	0.0573 (9)	0.0594 (9)	0.0700 (10)	0.0169 (7)	0.0187 (7)	0.0285 (8)
Na2	0.0671 (10)	0.0654 (10)	0.0815 (11)	0.0129 (8)	0.0205 (9)	0.0231 (9)
S1	0.0762 (7)	0.0715 (7)	0.0614 (6)	0.0100 (6)	0.0057 (5)	0.0314 (5)
S2	0.0701 (6)	0.0523 (6)	0.0648 (6)	0.0154 (5)	0.0086 (5)	0.0220 (5)
S3	0.0574 (6)	0.0833 (7)	0.0655 (6)	0.0198 (5)	0.0175 (5)	0.0343 (6)
S4	0.0627 (6)	0.0799 (7)	0.0688 (7)	0.0219 (6)	0.0221 (5)	0.0372 (6)
Cl1	0.1166 (11)	0.0807 (9)	0.1507 (14)	0.0348 (8)	0.0682 (10)	0.0316 (9)
Cl2	0.0950 (9)	0.1228 (11)	0.0983 (10)	0.0292 (8)	0.0479 (8)	0.0474 (9)
Cl3	0.0907 (9)	0.0727 (8)	0.1356 (13)	0.0064 (7)	0.0031 (9)	0.0048 (8)
Cl4	0.1041 (11)	0.0637 (8)	0.223 (2)	0.0245 (8)	0.0387 (12)	0.0141 (10)
O1	0.093 (2)	0.0693 (19)	0.108 (3)	0.0224 (18)	0.038 (2)	0.0427 (18)
O2	0.082 (2)	0.083 (2)	0.105 (3)	0.0229 (17)	0.0408 (19)	0.046 (2)
O3	0.0617 (18)	0.087 (2)	0.087 (2)	0.0267 (16)	0.0211 (15)	0.0370 (17)
O4	0.0539 (16)	0.0707 (18)	0.0757 (19)	0.0153 (14)	0.0110 (14)	0.0261 (15)
O5	0.078 (2)	0.079 (2)	0.085 (2)	0.0280 (17)	0.0244 (17)	0.0351 (18)
O11	0.106 (2)	0.0587 (17)	0.085 (2)	0.0095 (16)	0.0151 (18)	0.0311 (16)
O12	0.0725 (19)	0.078 (2)	0.095 (2)	0.0273 (16)	0.0029 (17)	0.0288 (17)
O13	0.101 (2)	0.088 (2)	0.0633 (19)	0.0288 (18)	0.0237 (17)	0.0277 (16)
O14	0.179 (4)	0.087 (3)	0.129 (3)	0.031 (3)	0.066 (3)	0.063 (2)
O15	0.093 (2)	0.133 (3)	0.062 (2)	0.020 (2)	0.0162 (18)	0.033 (2)
O16	0.078 (2)	0.191 (4)	0.107 (3)	0.066 (2)	0.047 (2)	0.087 (3)
C1	0.148 (6)	0.080 (4)	0.141 (6)	−0.001 (4)	0.071 (5)	0.046 (4)
C2	0.142 (6)	0.076 (4)	0.171 (7)	0.006 (4)	0.070 (5)	0.061 (4)
C3	0.066 (3)	0.121 (4)	0.088 (3)	0.039 (3)	0.033 (3)	0.060 (3)
C4	0.065 (3)	0.096 (4)	0.089 (3)	0.035 (3)	0.027 (2)	0.032 (3)
C5	0.081 (3)	0.069 (3)	0.082 (3)	0.023 (2)	0.014 (3)	0.020 (2)
C6	0.070 (3)	0.069 (3)	0.072 (3)	0.013 (2)	0.011 (2)	0.016 (2)
C7	0.054 (2)	0.088 (3)	0.079 (3)	0.021 (2)	0.012 (2)	0.031 (3)
C8	0.056 (3)	0.099 (4)	0.079 (3)	0.030 (2)	0.018 (2)	0.035 (3)
C9	0.107 (4)	0.065 (3)	0.116 (4)	0.038 (3)	0.044 (3)	0.027 (3)
C10	0.108 (4)	0.073 (3)	0.111 (4)	0.039 (3)	0.028 (3)	0.029 (3)
O6	0.089 (4)	0.074 (4)	0.064 (4)	0.023 (4)	0.023 (3)	0.018 (4)
O7	0.051 (4)	0.080 (5)	0.119 (6)	0.005 (4)	0.026 (4)	0.041 (4)
O8	0.079 (5)	0.094 (7)	0.121 (6)	0.044 (5)	0.042 (4)	0.039 (5)
O9	0.089 (5)	0.098 (5)	0.101 (5)	0.013 (5)	0.021 (4)	0.031 (5)
O10	0.123 (7)	0.082 (5)	0.108 (6)	0.018 (5)	0.024 (5)	0.023 (5)
C11	0.091 (8)	0.094 (10)	0.099 (11)	0.019 (8)	0.038 (8)	0.045 (8)
C12	0.076 (8)	0.079 (8)	0.085 (9)	−0.002 (6)	0.020 (7)	0.035 (7)
C13	0.093 (9)	0.056 (6)	0.135 (11)	0.007 (6)	0.035 (8)	0.016 (7)
C14	0.096 (10)	0.092 (12)	0.104 (10)	0.053 (9)	0.033 (7)	0.024 (8)
C15	0.086 (8)	0.123 (13)	0.091 (8)	0.058 (8)	0.042 (7)	0.055 (11)
C16	0.031 (5)	0.102 (8)	0.105 (10)	0.021 (6)	0.034 (6)	0.015 (7)
C17	0.112 (14)	0.066 (9)	0.127 (12)	−0.015 (8)	0.003 (12)	0.049 (8)
C18	0.068 (7)	0.080 (9)	0.092 (12)	−0.031 (7)	0.031 (7)	−0.010 (7)
C19	0.106 (10)	0.085 (10)	0.145 (12)	0.012 (8)	0.054 (10)	−0.003 (9)
C20	0.083 (10)	0.119 (12)	0.065 (8)	0.033 (8)	0.020 (8)	0.000 (7)
O6'	0.139 (8)	0.128 (11)	0.073 (6)	0.018 (7)	0.053 (6)	0.041 (7)
O7'	0.088 (7)	0.107 (8)	0.107 (7)	−0.005 (6)	0.021 (6)	0.036 (6)
O8'	0.106 (7)	0.076 (5)	0.114 (7)	0.026 (6)	0.038 (5)	0.035 (5)
O9'	0.068 (6)	0.102 (8)	0.110 (8)	−0.004 (5)	0.009 (5)	0.052 (6)
O10'	0.160 (11)	0.073 (6)	0.141 (9)	0.032 (7)	0.060 (8)	0.014 (6)
C11'	0.107 (15)	0.108 (14)	0.096 (14)	−0.006 (10)	0.003 (10)	0.060 (12)
C12'	0.095 (13)	0.099 (12)	0.104 (13)	−0.018 (11)	0.005 (10)	0.046 (10)
C13'	0.16 (3)	0.054 (10)	0.17 (2)	0.015 (12)	0.046 (17)	0.043 (13)
C14'	0.20 (2)	0.062 (10)	0.114 (16)	0.063 (15)	0.09 (2)	0.040 (11)
C15'	0.18 (2)	0.20 (2)	0.083 (14)	−0.104 (17)	−0.033 (13)	0.067 (15)
C16'	0.067 (8)	0.104 (14)	0.114 (14)	−0.022 (9)	−0.021 (8)	0.071 (12)
C17'	0.29 (4)	0.19 (2)	0.061 (14)	0.16 (3)	0.011 (18)	0.036 (15)
C18'	0.20 (2)	0.060 (9)	0.096 (11)	0.043 (12)	0.046 (13)	−0.001 (8)
C19'	0.073 (11)	0.118 (14)	0.075 (11)	0.031 (9)	0.010 (8)	0.020 (10)
C20'	0.102 (10)	0.120 (14)	0.083 (10)	0.004 (12)	0.033 (9)	0.039 (11)
C21	0.072 (3)	0.074 (3)	0.063 (3)	0.011 (2)	0.013 (2)	0.025 (2)
C22	0.088 (3)	0.075 (3)	0.087 (3)	0.009 (3)	0.020 (3)	0.031 (3)
C23	0.072 (3)	0.130 (5)	0.091 (4)	0.025 (3)	0.028 (3)	0.058 (3)
C24	0.086 (4)	0.151 (5)	0.094 (4)	0.029 (4)	0.034 (3)	0.067 (4)

### Geometric parameters (Å, °)

**Table:**

Na1—O1	2.417 (3)	O7—C12	1.414 (9)
Na1—O2	2.432 (3)	O8—C15	1.414 (9)
Na1—O4	2.442 (3)	O8—C14	1.431 (10)
Na1—O13	2.447 (3)	O9—C17	1.396 (9)
Na1—O5	2.452 (3)	O9—C16	1.413 (8)
Na1—O3	2.482 (3)	O10—C18	1.412 (8)
Na1—O12	2.732 (4)	O10—C19	1.415 (9)
Na1—S2	3.110 (2)	C11—C12	1.503 (9)
Na2—O15	2.305 (4)	C11—H11A	0.9700
Na2—O8'	2.389 (7)	C11—H11B	0.9700
Na2—O10	2.390 (7)	C12—H12A	0.9700
Na2—O6	2.396 (6)	C12—H12B	0.9700
Na2—O9	2.415 (7)	C13—C14	1.498 (10)
Na2—O6'	2.434 (8)	C13—H13A	0.9700
Na2—O7	2.439 (6)	C13—H13B	0.9700
Na2—O9'	2.457 (7)	C14—H14A	0.9700
Na2—O8	2.466 (6)	C14—H14B	0.9700
Na2—O7'	2.471 (8)	C15—C16	1.515 (9)
Na2—O10'	2.482 (8)	C15—H15A	0.9700
Na2—C19	3.084 (13)	C15—H15B	0.9700
S1—C21	1.742 (5)	C16—H16A	0.9700
S1—S2	2.1367 (17)	C16—H16B	0.9700
S2—O12	1.428 (3)	C17—C18	1.507 (10)
S2—O11	1.432 (3)	C17—H17A	0.9700
S2—O13	1.449 (3)	C17—H17B	0.9700
S3—C23	1.734 (5)	C18—H18A	0.9700
S3—S4	2.1368 (16)	C18—H18B	0.9700
S4—O14	1.416 (4)	C19—C20	1.501 (10)
S4—O15	1.417 (3)	C19—H19A	0.9700
S4—O16	1.419 (3)	C19—H19B	0.9700
Cl1—C22	1.715 (5)	C20—H20A	0.9700
Cl2—C21	1.768 (5)	C20—H20B	0.9700
Cl3—C24	1.742 (6)	O6'—C20'	1.407 (9)
Cl4—C23	1.830 (6)	O6'—C11'	1.413 (10)
O1—C10	1.401 (5)	O7'—C12'	1.406 (9)
O1—C1	1.402 (6)	O7'—C13'	1.437 (10)
O2—C2	1.398 (6)	O8'—C14'	1.405 (9)
O2—C3	1.407 (5)	O8'—C15'	1.415 (10)
O3—C4	1.406 (5)	O9'—C16'	1.413 (10)
O3—C5	1.409 (5)	O9'—C17'	1.430 (10)
O4—C7	1.408 (5)	O10'—C18'	1.403 (9)
O4—C6	1.416 (5)	O10'—C19'	1.410 (10)
O5—C8	1.407 (5)	C11'—C12'	1.510 (10)
O5—C9	1.422 (5)	C11'—H11C	0.9700
C1—C2	1.420 (6)	C11'—H11D	0.9700
C1—H1A	0.9700	C12'—H12C	0.9700
C1—H1B	0.9700	C12'—H12D	0.9700
C2—H2A	0.9700	C13'—C14'	1.529 (10)
C2—H2B	0.9700	C13'—H13C	0.9700
C3—C4	1.509 (6)	C13'—H13D	0.9700
C3—H3A	0.9700	C14'—H14C	0.9700
C3—H3B	0.9700	C14'—H14D	0.9700
C4—H4A	0.9700	C15'—C16'	1.510 (10)
C4—H4B	0.9700	C15'—H15C	0.9700
C5—C6	1.509 (6)	C15'—H15D	0.9700
C5—H5A	0.9700	C16'—H16C	0.9700
C5—H5B	0.9700	C16'—H16D	0.9700
C6—H6A	0.9700	C17'—C18'	1.508 (10)
C6—H6B	0.9700	C17'—H17C	0.9700
C7—C8	1.520 (6)	C17'—H17D	0.9700
C7—H7A	0.9700	C18'—H18C	0.9700
C7—H7B	0.9700	C18'—H18D	0.9700
C8—H8A	0.9700	C19'—C20'	1.518 (10)
C8—H8B	0.9700	C19'—H19C	0.9700
C9—C10	1.520 (7)	C19'—H19D	0.9700
C9—H9A	0.9700	C20'—H20C	0.9700
C9—H9B	0.9700	C20'—H20D	0.9700
C10—H10A	0.9700	C21—C22	1.282 (6)
C10—H10B	0.9700	C22—H22A	0.9300
O6—C11	1.411 (8)	C23—C24	1.241 (6)
O6—C20	1.419 (9)	C24—H24A	0.9300
O7—C13	1.402 (8)		
			
O1—Na1—O2	68.31 (12)	O1—C10—C9	107.7 (4)
O1—Na1—O4	137.10 (12)	O1—C10—H10A	110.2
O2—Na1—O4	133.84 (13)	C9—C10—H10A	110.2
O1—Na1—O13	118.23 (13)	O1—C10—H10B	110.2
O2—Na1—O13	99.92 (12)	C9—C10—H10B	110.2
O4—Na1—O13	95.82 (11)	H10A—C10—H10B	108.5
O1—Na1—O5	69.23 (12)	C11—O6—C20	111.8 (10)
O2—Na1—O5	121.52 (12)	C11—O6—Na2	112.6 (7)
O4—Na1—O5	68.16 (11)	C20—O6—Na2	114.0 (8)
O13—Na1—O5	135.41 (12)	C13—O7—C12	114.7 (10)
O1—Na1—O3	127.39 (12)	C13—O7—Na2	104.8 (7)
O2—Na1—O3	68.27 (12)	C12—O7—Na2	108.1 (7)
O4—Na1—O3	66.85 (11)	C15—O8—C14	104.7 (10)
O13—Na1—O3	97.24 (11)	C15—O8—Na2	118.9 (7)
O5—Na1—O3	112.06 (11)	C14—O8—Na2	116.4 (7)
O1—Na1—O12	93.38 (11)	C17—O9—C16	115.2 (9)
O2—Na1—O12	137.70 (12)	C17—O9—Na2	110.1 (8)
O4—Na1—O12	85.79 (10)	C16—O9—Na2	113.8 (6)
O13—Na1—O12	54.35 (10)	C18—O10—C19	118.2 (10)
O5—Na1—O12	82.33 (10)	C18—O10—Na2	117.8 (7)
O3—Na1—O12	139.14 (11)	C19—O10—Na2	105.4 (7)
O1—Na1—S2	107.27 (10)	O6—C11—C12	113.0 (12)
O2—Na1—S2	120.56 (10)	O6—C11—H11A	109.0
O4—Na1—S2	91.16 (8)	C12—C11—H11A	109.0
O13—Na1—S2	27.02 (7)	O6—C11—H11B	109.0
O5—Na1—S2	109.18 (9)	C12—C11—H11B	109.0
O3—Na1—S2	119.74 (9)	H11A—C11—H11B	107.8
O12—Na1—S2	27.34 (6)	O7—C12—C11	105.2 (10)
O15—Na2—O8'	116.7 (2)	O7—C12—H12A	110.7
O15—Na2—O10	107.5 (2)	C11—C12—H12A	110.7
O8'—Na2—O10	107.4 (3)	O7—C12—H12B	110.7
O15—Na2—O6	89.46 (18)	C11—C12—H12B	110.7
O8'—Na2—O6	151.6 (3)	H12A—C12—H12B	108.8
O10—Na2—O6	72.0 (2)	O7—C13—C14	112.1 (11)
O15—Na2—O9	143.1 (2)	O7—C13—Na2	49.4 (5)
O8'—Na2—O9	39.7 (3)	C14—C13—Na2	86.1 (8)
O10—Na2—O9	70.4 (2)	O7—C13—H13A	109.2
O6—Na2—O9	122.4 (2)	C14—C13—H13A	109.2
O15—Na2—O6'	88.3 (3)	Na2—C13—H13A	80.2
O8'—Na2—O6'	134.8 (4)	O7—C13—H13B	109.2
O10—Na2—O6'	98.8 (3)	C14—C13—H13B	109.2
O6—Na2—O6'	27.7 (3)	Na2—C13—H13B	158.2
O9—Na2—O6'	128.6 (3)	H13A—C13—H13B	107.9
O15—Na2—O7	101.9 (2)	O8—C14—C13	105.8 (11)
O8'—Na2—O7	92.7 (3)	O8—C14—H14A	110.6
O10—Na2—O7	130.9 (3)	C13—C14—H14A	110.6
O6—Na2—O7	69.8 (2)	O8—C14—H14B	110.6
O9—Na2—O7	106.3 (3)	C13—C14—H14B	110.6
O6'—Na2—O7	43.4 (3)	H14A—C14—H14B	108.7
O15—Na2—O9'	125.0 (2)	O8—C15—C16	106.0 (9)
O8'—Na2—O9'	67.9 (3)	O8—C15—H15A	110.5
O10—Na2—O9'	39.5 (3)	C16—C15—H15A	110.5
O6—Na2—O9'	107.3 (3)	O8—C15—H15B	110.5
O9—Na2—O9'	33.6 (3)	C16—C15—H15B	110.5
O6'—Na2—O9'	129.1 (3)	H15A—C15—H15B	108.7
O7—Na2—O9'	133.1 (3)	O9—C16—C15	103.3 (10)
O15—Na2—O8	106.7 (2)	O9—C16—H16A	111.1
O8'—Na2—O8	28.6 (3)	C15—C16—H16A	111.1
O10—Na2—O8	134.5 (3)	O9—C16—H16B	111.1
O6—Na2—O8	137.0 (3)	C15—C16—H16B	111.1
O9—Na2—O8	64.2 (2)	H16A—C16—H16B	109.1
O6'—Na2—O8	111.4 (4)	O9—C17—C18	118.6 (11)
O7—Na2—O8	68.0 (3)	O9—C17—H17A	107.7
O9'—Na2—O8	95.6 (3)	C18—C17—H17A	107.7
O15—Na2—O7'	110.4 (2)	O9—C17—H17B	107.7
O8'—Na2—O7'	69.3 (3)	C18—C17—H17B	107.7
O10—Na2—O7'	138.6 (3)	H17A—C17—H17B	107.1
O6—Na2—O7'	92.2 (3)	O10—C18—C17	105.5 (10)
O9—Na2—O7'	88.4 (3)	O10—C18—H18A	110.6
O6'—Na2—O7'	66.8 (4)	C17—C18—H18A	110.6
O7—Na2—O7'	23.5 (3)	O10—C18—H18B	110.6
O9'—Na2—O7'	120.5 (3)	C17—C18—H18B	110.6
O8—Na2—O7'	45.0 (4)	H18A—C18—H18B	108.8
O15—Na2—O10'	112.3 (3)	O10—C19—C20	118.4 (14)
O8'—Na2—O10'	126.2 (4)	O10—C19—Na2	48.4 (6)
O10—Na2—O10'	34.3 (3)	C20—C19—Na2	82.3 (8)
O6—Na2—O10'	40.8 (3)	O10—C19—H19A	107.7
O9—Na2—O10'	87.0 (3)	C20—C19—H19A	107.7
O6'—Na2—O10'	65.4 (4)	Na2—C19—H19A	91.5
O7—Na2—O10'	98.1 (3)	O10—C19—H19B	107.7
O9'—Na2—O10'	66.5 (4)	C20—C19—H19B	107.7
O8—Na2—O10'	140.7 (4)	Na2—C19—H19B	154.3
O7'—Na2—O10'	112.8 (4)	H19A—C19—H19B	107.1
O15—Na2—C19	97.5 (3)	O6—C20—C19	101.2 (10)
O8'—Na2—C19	132.2 (3)	O6—C20—H20A	111.5
O10—Na2—C19	26.3 (2)	C19—C20—H20A	111.5
O6—Na2—C19	46.6 (3)	O6—C20—H20B	111.5
O9—Na2—C19	93.1 (3)	C19—C20—H20B	111.5
O6'—Na2—C19	74.1 (3)	H20A—C20—H20B	109.3
O7—Na2—C19	112.8 (3)	C20'—O6'—C11'	110.7 (12)
O9'—Na2—C19	65.2 (3)	C20'—O6'—Na2	118.8 (9)
O8—Na2—C19	155.2 (4)	C11'—O6'—Na2	115.6 (10)
O7'—Na2—C19	130.3 (4)	C12'—O7'—C13'	107.0 (13)
O10'—Na2—C19	18.3 (4)	C12'—O7'—Na2	103.6 (10)
C21—S1—S2	100.39 (15)	C13'—O7'—Na2	101.2 (11)
O12—S2—O11	116.0 (2)	C14'—O8'—C15'	127.3 (16)
O12—S2—O13	111.5 (2)	C14'—O8'—Na2	111.8 (9)
O11—S2—O13	115.70 (19)	C15'—O8'—Na2	113.5 (11)
O12—S2—S1	106.39 (14)	C16'—O9'—C17'	105.4 (11)
O11—S2—S1	99.14 (14)	C16'—O9'—Na2	114.6 (8)
O13—S2—S1	106.32 (14)	C17'—O9'—Na2	104.8 (12)
O12—S2—Na1	61.45 (14)	C18'—O10'—C19'	112.4 (13)
O11—S2—Na1	141.11 (14)	C18'—O10'—Na2	112.7 (9)
O13—S2—Na1	50.06 (13)	C19'—O10'—Na2	109.8 (11)
S1—S2—Na1	119.20 (6)	O6'—C11'—C12'	104.8 (14)
C23—S3—S4	100.79 (17)	O6'—C11'—H11C	110.8
O14—S4—O15	112.8 (3)	C12'—C11'—H11C	110.8
O14—S4—O16	116.0 (3)	O6'—C11'—H11D	110.8
O15—S4—O16	114.3 (2)	C12'—C11'—H11D	110.8
O14—S4—S3	106.64 (17)	H11C—C11'—H11D	108.9
O15—S4—S3	105.45 (15)	O7'—C12'—C11'	103.9 (17)
O16—S4—S3	99.79 (15)	O7'—C12'—Na2	50.4 (8)
C10—O1—C1	115.6 (4)	C11'—C12'—Na2	83.3 (11)
C10—O1—Na1	115.0 (3)	O7'—C12'—H12C	111.0
C1—O1—Na1	115.6 (3)	C11'—C12'—H12C	111.0
C2—O2—C3	111.5 (4)	Na2—C12'—H12C	160.1
C2—O2—Na1	114.7 (3)	O7'—C12'—H12D	111.0
C3—O2—Na1	114.8 (3)	C11'—C12'—H12D	111.0
C4—O3—C5	114.4 (3)	Na2—C12'—H12D	76.7
C4—O3—Na1	104.6 (3)	H12C—C12'—H12D	109.0
C5—O3—Na1	104.4 (3)	O7'—C13'—C14'	101.0 (14)
C7—O4—C6	112.6 (3)	O7'—C13'—Na2	51.6 (8)
C7—O4—Na1	115.3 (2)	C14'—C13'—Na2	79.5 (10)
C6—O4—Na1	116.1 (2)	O7'—C13'—H13C	111.6
C8—O5—C9	112.7 (4)	C14'—C13'—H13C	111.6
C8—O5—Na1	105.6 (2)	Na2—C13'—H13C	77.1
C9—O5—Na1	105.5 (3)	O7'—C13'—H13D	111.6
S2—O12—Na1	91.22 (16)	C14'—C13'—H13D	111.6
S2—O13—Na1	102.92 (17)	Na2—C13'—H13D	162.5
S4—O15—Na2	148.2 (2)	H13C—C13'—H13D	109.4
O1—C1—C2	116.3 (5)	O8'—C14'—C13'	113.1 (16)
O1—C1—H1A	108.2	O8'—C14'—H14C	109.0
C2—C1—H1A	108.2	C13'—C14'—H14C	109.0
O1—C1—H1B	108.2	O8'—C14'—H14D	109.0
C2—C1—H1B	108.2	C13'—C14'—H14D	109.0
H1A—C1—H1B	107.4	H14C—C14'—H14D	107.8
O2—C2—C1	112.8 (5)	O8'—C15'—C16'	115.5 (14)
O2—C2—H2A	109.0	O8'—C15'—H15C	108.4
C1—C2—H2A	109.0	C16'—C15'—H15C	108.4
O2—C2—H2B	109.0	O8'—C15'—H15D	108.4
C1—C2—H2B	109.0	C16'—C15'—H15D	108.4
H2A—C2—H2B	107.8	H15C—C15'—H15D	107.5
O2—C3—C4	107.9 (4)	O9'—C16'—C15'	101.1 (15)
O2—C3—H3A	110.1	O9'—C16'—H16C	111.5
C4—C3—H3A	110.1	C15'—C16'—H16C	111.5
O2—C3—H3B	110.1	O9'—C16'—H16D	111.5
C4—C3—H3B	110.1	C15'—C16'—H16D	111.5
H3A—C3—H3B	108.4	H16C—C16'—H16D	109.4
O3—C4—C3	106.9 (4)	O9'—C17'—C18'	115.5 (14)
O3—C4—H4A	110.3	O9'—C17'—H17C	108.4
C3—C4—H4A	110.3	C18'—C17'—H17C	108.4
O3—C4—H4B	110.3	O9'—C17'—H17D	108.4
C3—C4—H4B	110.3	C18'—C17'—H17D	108.4
H4A—C4—H4B	108.6	H17C—C17'—H17D	107.5
O3—C5—C6	105.6 (4)	O10'—C18'—C17'	89.7 (15)
O3—C5—H5A	110.6	O10'—C18'—H18C	113.7
C6—C5—H5A	110.6	C17'—C18'—H18C	113.7
O3—C5—H5B	110.6	O10'—C18'—H18D	113.7
C6—C5—H5B	110.6	C17'—C18'—H18D	113.7
H5A—C5—H5B	108.8	H18C—C18'—H18D	110.9
O4—C6—C5	107.2 (3)	O10'—C19'—C20'	104.7 (13)
O4—C6—H6A	110.3	O10'—C19'—H19C	110.8
C5—C6—H6A	110.3	C20'—C19'—H19C	110.8
O4—C6—H6B	110.3	O10'—C19'—H19D	110.8
C5—C6—H6B	110.3	C20'—C19'—H19D	110.8
H6A—C6—H6B	108.5	H19C—C19'—H19D	108.9
O4—C7—C8	108.1 (3)	O6'—C20'—C19'	108.7 (15)
O4—C7—H7A	110.1	O6'—C20'—H20C	110.0
C8—C7—H7A	110.1	C19'—C20'—H20C	110.0
O4—C7—H7B	110.1	O6'—C20'—H20D	110.0
C8—C7—H7B	110.1	C19'—C20'—H20D	110.0
H7A—C7—H7B	108.4	H20C—C20'—H20D	108.3
O5—C8—C7	107.2 (4)	C22—C21—S1	127.3 (4)
O5—C8—H8A	110.3	C22—C21—Cl2	116.0 (4)
C7—C8—H8A	110.3	S1—C21—Cl2	116.6 (3)
O5—C8—H8B	110.3	C21—C22—Cl1	123.1 (4)
C7—C8—H8B	110.3	C21—C22—H22A	118.4
H8A—C8—H8B	108.5	Cl1—C22—H22A	118.4
O5—C9—C10	107.5 (4)	C24—C23—S3	131.6 (5)
O5—C9—H9A	110.2	C24—C23—Cl4	110.9 (5)
C10—C9—H9A	110.2	S3—C23—Cl4	117.3 (3)
O5—C9—H9B	110.2	C23—C24—Cl3	117.1 (5)
C10—C9—H9B	110.2	C23—C24—H24A	121.5
H9A—C9—H9B	108.5	Cl3—C24—H24A	121.5
